# Synergistic effect of PARP inhibitor and BRD4 inhibitor in multiple models of ovarian cancer

**DOI:** 10.1111/jcmm.17683

**Published:** 2023-02-08

**Authors:** Yuhan Huang, Chen Liu, Lixin You, Xi Li, Gang Chen, Junpeng Fan

**Affiliations:** ^1^ Department of Obstetrics and Gynecology Tongji Hospital, Tongji Medical College, Huazhong University of Science and Technology Wuhan China; ^2^ National Clinical Research Center for Obstetrics and Gynecology Cancer Biology Research Center (Key Laboratory of the Ministry of Education), Tongji Hospital, Tongji Medical College, Huazhong University of Science and Technology Wuhan China; ^3^ Department of Obstetrics and Gynecology Shanghai General Hospital, Shanghai Jiao Tong University School of Medicine Shanghai China

**Keywords:** AZD5153, olaparib, ovarian cancer, PDX, synergistic activity

## Abstract

Ovarian cancer has the highest facility rate among gynaecological tumours. Current therapies including PARP inhibitors have a defect that ovarian tumour is easy to recurrent and become resistant to therapy. To solve this problem, we found that BRD4 inhibitor AZD5153 and PARP inhibitor olaparib had a widespread synergistic effect in multiple models with different gene backgrounds. AZD5153 sensitizes cells to olaparib and reverses the acquired resistance by down‐regulating PTEN expression levels to destabilize hereditary materials. In this study, we used the following multiple ovarian cancer models PDX, PDO and 3D/2D cell lines to elucidate the co‐effect of AZD5153 and olaparib in vivo and in vitro. The similar results of these models further proved that the mechanism identified was consistent with the biological process occurring in ovarian cancer patients after drug treatment. This consistency between the results of different models suggests the possibility of translating these laboratory research findings into clinical studies towards developing treatments.

## 1 INTRODUCTION

Ovarian cancer is the leading cause of cancer mortality among patients with gynaecological tumours.^1^ In the past few years, new approaches have been developed to treat ovarian tumours. However, since 2017 to date, the mortality rate of ovarian cancer has shown no sign of decreasing, which demonstrates the treatment challenges. The refractoriness of ovarian cancer results from the high incidence of recurrence and drug resistance.^2^, ^3^ Clinical trials have shown that the poly‐ADP ribose polymerase (PARP) inhibitors could improve the progression‐free survival (PFS) of ovarian cancer patients.^4^, ^5^ However, the wide use of olaparib as a single agent inevitably induce resistance. Therefore, it is urgent to find solutions to delay or even reverse olaparib resistance. In recent years, some reports have found that Bromodomain and extra‐terminal motif (BET) inhibition may be a potential strategy to reverse PARP inhibitors resistance.^6^, ^7^ The effectiveness of the combined effect of PARP and BET inhibition has been verified in different cancers.,^8^, ^9^, ^10^ and our previous study in ovarian cancer showed that combining the PARP inhibitor BMN673 with the BET inhibitor JQ1 resulted in a potent lethal anticancer effect.^11^

The patient‐derived organoids (PDO) model is a three‐dimensional (3D) model in which human cancer tissue is cultured in vitro. Because the PDO model can be used to examine drug activity in primary tumours, it could be beneficial to translational medicine studies^12^, ^13^ and have already been used to explore drug effects in ovarian cancer.^14^ The patient‐derived xenografts (PDX) model is another in vivo model that is widely used in precision medicine research,^15^, ^16^, ^17^ and it retains the microenvironment and heterogeneity of the primary tumour.^18^ The joint application of PDO and PDX in drug screening and mechanism exploration can be used in models to provide a closer simulation of the condition of patients.^19^

In this study, we choose olaparib and AZD5153, respectively, as representative agents to further investigate the synergistic effects of PARP and BET inhibitors. olaparib has been confirmed to show less toxicity and off‐target effects clinically^20^, ^21^ and it can be used at a high dose to achieve maximal PARP‐inhibiting effect.^22^, ^23^ AZD5153 is a more specific small molecular inhibitor of the BET protein bromodomain‐containing protein 4 (BRD4).^8^, ^24^ The experimental models we selected were PDO and PDX, which mimic the tumour environment in patients. We not only used these models to investigate the actions of these drugs, but we also explored the mechanisms underlying these actions using experiments that are usually conducted in cell lines.

## 2 METHODS

### 2.1 Cell lines and cell culture

Human ovarian cancer cell lines (HOC7, OVCAR8) were obtained from MDACC characterized Cell line Core Facility. Human ovarian cell lines (A2780, ES2, SKOV3, OVCAR3, Caov3, OV90, TOV‐112D and TOV‐21G) were obtained from the American Type Culture Collection (ATCC). ID8 is a mouse ovarian cancer cell line derived from C57BL/6, which was a gift by Professor K. Roby (Department of Anatomy and Cell Biology, University of Kansas, USA).

Cell lines were all passaged less than 30 times and were cultured under 37°C, 5% CO_2_ incubator. 3D cell was cultured in the same condition as PDO models.

### 2.2 Antibodies and compounds

Olaparib (S1060), AZD5153 (S8344) were bought from Sellek. IdU (1336001) and CIdU (C6891) were from Sigma. CELLTiter GLO 3D (G9682) were from PROMEGA. Components added in the PDO culture medium are all bought from BD Biosciences. GAPDH (A19056), β‐TUBULIN (AC008), α‐TUBULIN (A6830), RFC4 (A5485) and SMC1A (A4693) antibodies were from Abclonal. PTEN (ab267787), RFC3 (ab182143), P‐SMC1A (ab75768), RAD51 (ab133534), P‐CHK317 (ab226929), PI3K (ab40776), BRCA1 (ab238983) and BRCA2 (ab239375) antibodies were from Abcam. Brd4 (#83375), γH2AX (#80312, #9718), RPA32 (#52448) and P‐RPA32 (#83745) antibodies were from Cell Signaling Technology (CST).

### 2.3 Clinical specimens

All primary ovarian cancer tissues are anonymized and obtained from Tongji Hospital, Tongji Medical College, Huazhong University of Science and Technology, Wuhan, China, in accordance with the Declaration of Helsinki. All the operations were approved by the Ethics or Institutional Review Board.

### 2.4 Establishment of PDX model

PDX (Patient‐derived xenografts) models were obtained by subcutaneously transplanting fresh tumour tissue into nude mice. 6–8 weeks‐old female BALB/C nu‐mice were purchased from Beijing HFK Bioscience and raise in specific pathogen‐free conditions. All manipulations were performed under the guidance of the Animal Laboratory of Tongji Hospital. Solid tumour xenografts are passaged with the same technique after being established.

### 2.5 Establishment of PDO model

PDO (Patient‐derived organoids) models were established by using patient tissues from Tongji Hospital. Fresh tumour tissue can be stored in DMEM/F12 (1% PS) under 4°C within 12 h. The tumour tissue was minced and filtered through 70um (Falcon, #352360) and 40um (Falcon, #352340) strainer, to get a suspension of multicell‐spheroid, which has a diameter between 40‐100um. After being treated with red cell lysis buffer and washed with PBS, the multicell‐spheroid was resuspended by Matrigel (Corning, #356231). The suspension was planted into 6‐ or 96‐well plates according to the following experiments. For example, 10 μL Matrigel with 5000 organoids per well was used for short drug screening. The Matrigel needs to solidify at 37°C for 30 min, then the culture medium was added to the plates.

PDO was cultured in DMEM/F12 with Glutanmax 1×, HEPES 1×, R‐spondin 100 ng/mL, noggin 100 ng/mL, EGF 50 ng/mL, FGF10 10 ng/mL, FGF2 10 ng/mL, B27 50×, nicotinamide 10 mmoL/mL, N‐Acetylglycine 25 mmoL/mL, prostaglandin E2 1 umol/ml, SB02190 10 umolg/ml, A8301 500 nmoL/mL and Y27632 10 μmoL/mL.

### 2.6 Generation of PARP inhibitor resistant cells

A2780, HOC7 and ID8 cells were cultured with an increased concentration of olaparib. After 3–4 months of treatment, these cells can grow rapidly in the presence of 10 uM olaparib. Cells were cultured in the absence of olaparib for 1 month. Before use, IC50 was calculated again to confirm its drug resistance.

### 2.7 Alkaline single‐cell agarose gel electrophoresis (Comet) assay

Alkaline comet assay was performed by following the manufacturer's instructions of Trevigin's Comet Assay Kit (#4250‐050‐K). Cells were suspended in Low melting agarose and mounted on comet slides as instructed. After the gel was solidified, the comet slide was incubated in lysis solution for 1 h at 4°C and the freshly prepared unwinding solution for 20 min at room temperature in a dark place. Electrophoresis was performed under 21 V for 25 min in the freshly prepared electrophoresis solution. Slides can be stained with SYBR Green I to analyse the comet tail, which stands for DNA strand breakage. Tail length of the comet tail was calculated by using CASP (Comet Assay Software Project), and shown by the relative amount compared with control. Average damage from three independent experiments was calculated.

### 2.8 DNA fibre assay

Cells were labelled with 25uM CIdU for 30 min, washed by PBS for 3 times, and then labelled with 250uM IdU for 45 min. After labelling, cells were collected, resuspended to 5 × 10^5^ cells/ml in ice‐cold PBS. 7ul freshly prepared spreading buffer was mixed with 2ul cell suspension and pipetted onto a microscope slide. By carefully tilt the slides at 25–60 degrees, the stream of DNA was allowed to travel slowly down the slide. Then the slides were airdried and fixed in methanol/acetic acid (3:1) for 10 min. Slides were then washed with ddH_2_O, denatured in 2 N HCL for 30 min, and block with 5% BSA‐PBS. After that, the slides were incubated with 1:150 rat anti‐BrdU (Abcam, ab6326) and 1:50 mouse anti‐BrdU (BD Biosciences, #347580) antibody for 3 h at room temperature. Then the slides were rinsed and incubated in 1:150 anti‐Rat AlexaFluor 488 antibody and 1:150 anti‐Mouse AlexaFluor 568 antibody for 1 h at room temperature. The results were obtained by using Zeiss Laser Scanning Confocal Microscope 880.

### 2.9 Fluorescence in situ hybridization (FISH) assay

The slides were pretreated with xylene and gradient ethanol to dewaxing and hydration, boiled, digest with 200 μL pepsin solution, and then put into 2×SSC at room temperature for 3 min. Dehydration with gradient ethanol for 2 times and dried at room temperature. Samples and probes were hybridized in an environment protected from light. Then the slides were washed and counterstained.

The probes (LBP Guangzhou, # F.01005‐01) can hybrid with chromosome 10 centromere (green signal) and PTEN gene (red signal). The normal cell contained two red and two green signals. A PTEN amplificated signal mode contains more red signals.

### 2.10 Metaphase spread assay

Cells were planted in six‐well plates, and cultured till sub‐culture at 70% confluency. Cells were exposed to colchicine (100 ng/mL) (Sellek, S2284) for 3 h, collected and resuspension in hypotonic solution (0.075 M KCl) for 30 min at 37°C incubator. Cells were then fixed in methanol: acetic acid (3:1) at 4°C for 30 min and repeat for 3 times. Then the fixed cells were dropped on precooled slides and put into a 65°C incubator to air‐dried. After cooled down, the slides were stained in 3% Giemsa and coded for blind analysis. A total of 25 metaphases was analysed from each sample to detect the presence of chromosomal fragments.

### 2.11 NCI60, CCLE and GSCALite

Gene expression profiles (Gene transcript level z score) for correlations analysis in NCI60 human tumour cell lines were obtained using the web‐based tool provided by CellMiner (http://discover.nci.nih.gov/cellminer/).

Gene mutation data of cell lines were collected from Cancer Cell Line Encyclopedia (CCLE) (https://portals.broadinstitute.org/ccle/data/browseData?conversationPropagation=begin).

The correlation between gene expression and drug reaction of human tumour cell lines was obtained by using the web‐based tool provided by GSCALite (http://bioinfo.life.hust.edu.cn/web/GSCALite/). The gene expression and drug reaction data were collected by GSCALite from CCLE, CTRP and GDSC.

### 2.12 ChIP‐Seq analysis

ChIP‐seq data for human cell lines from PMID 27803105, PMID 29491412 and GSM2090919, GSM 2090922 was collected from Cistrome (http://cistrome.org/db/#/) and analysed by UCSC genome browser (http://genome.ucsc.edu/).

### 2.13 Crispr screening and mass spectrum data

Crispr screening data of olaparib‐acquired resistant cell lines were downloaded from PMID29973717 and the mass‐spectrum data (ID 013196) was downloaded from proteome‐central (http://proteomecentral.proteomexchange.org/cgi/GetDataset?ID=PXD013196). The pathway enrichment of the data was done by using web‐based tool provided by DAVID (https://david.ncifcrf.gov/summary.jsp).

### 2.14 Q‐RTPCR

Total RNA (1 μg) was reversely transcribed into cDNA with the Hiscript Q‐RT SuperMix for qPCR (Vazyme Biotech). According to the manufacturer's instructions. Real‐Time PCR Master Mixes kit (Life Technologies) was used for the thermocycling reaction in a BioRad CFX96 Real‐Time system. The mRNA levels analysis was carried out in triplicate and normalized by GAPDH. Primers sequences of PTEN were as listed.

Mouse: Forward, TGGATTCGACTTAGACTTGACCT; Reverse, GCGGTGTCATAATGTCTCTCAG; Human: Forward, TGGATTCGACTTAGACTTGACCT; Reverse, GGTGGGTTATGGTCTTCAAAAGG.

### 2.15 Virus transfection protocol

In a six‐well culture plate, cells are cultured at 50%–70% confluency in an antibiotic‐free growth medium supplemented with FBS. The mixture was gently mixed and incubated for 30 min at room temperature. Use the antibiotic‐free growth medium to wash the cells twice. Add 0.2 mL virus to well for each transfection. Add 40 uL Transfection Reagent Complex to well, covering the entire layer and gently swirling the plate. Incubate the cells at 37°C in a CO_2_ incubator for 6 h. Add 1 mL normal growth medium containing 2 times FBS and antibiotics into each well, and incubate for 18–24 h in 37°C, 5% CO_2_ incubator. Use puromycin to select stably transfected cells. PTEN shRNA and PTEN overexpression virus are bought from Genechem.

### 2.16 Western blot assay

Cells were lysed with RIPA buffer (Servicebio, G2002‐100) containing protease and phosphatase inhibitor cocktail (Servicebio, G2002‐100). The lysates were centrifuged at 12000 rpm 4°C for 20 min to collect supernatants. After determining the protein content, the cell lysates were separated by 10% SDS‐PAGE and electro‐transferred onto 0.45 um PVDF membranes. The membranes were blocked with 5% BSA‐TBST at room temperature and then incubated with primary antibodies at 4°C overnight. Secondary antibody (Antgene) was incubated for 1 h at room temperature. Bands were visualized by using WesternBright ECL Kit (Advansta, 190113‐13).

### 2.17 Immunohistochemistry

Immunohistochemistry was performed as previously described.^11^ The primary antibody included Ki67 (Abcam, 1:500), RAD51 (Abcam, 1:500) and γH2AX (Abcam, 1:500).

Tumour cell staining was assigned a score using a semi‐quantitative grading system: 0, 0%–5% tumour‐cell staining; 1, 5%–25% tumour‐cell staining; 2, 26%–50% tumour‐cell staining; 3, 51%–75% tumour‐cell staining; and 4, >75% tumour‐cell staining. Staining intensity was assigned a score using a semi‐quantitative four‐category grading system: 0, no staining; 1, weak staining; 2, moderate staining; and 3, strong staining. Every core was assessed individually and the mean of three readings was calculated for every case. The tumour cell staining score was determined separately by two independent experts simultaneously under the same conditions. In rare cases, discordant scores were reevaluated and scored based on consensus opinion.

### 2.18 In vivo small animal imaging technology

C57BL/6 mice were purchased from Beijing HFK Bioscience and raise in specific pathogen‐free conditions. ID8 cells are planted intraperitoneally into mice.

Mice were anaesthetised intraperitoneally with 4% chloral hydrate (g / ml) at a dose of 150 ul/20 g body weight. 5 mg of the fluorescent substrate was injected intraperitoneally for 10 min. The anaesthetised and injected mice were placed in the instrument. The images were collected by the small animal imaging instrument (SIEMENS Inveon), and the same low and high values of fluorescence signals were set for each group.

### 2.19 Statistical analysis

GraphPad Prism 8 and IBM SPSS statistic 26.0 were used for statistical analysis. *p* < 0.05 was considered to indicate a significant difference.

## 3 RESULTS

### 3.1 Olaparib treatment can increase the expression of PTEN which relates to olaparib resistance

**FIGURE 1:**
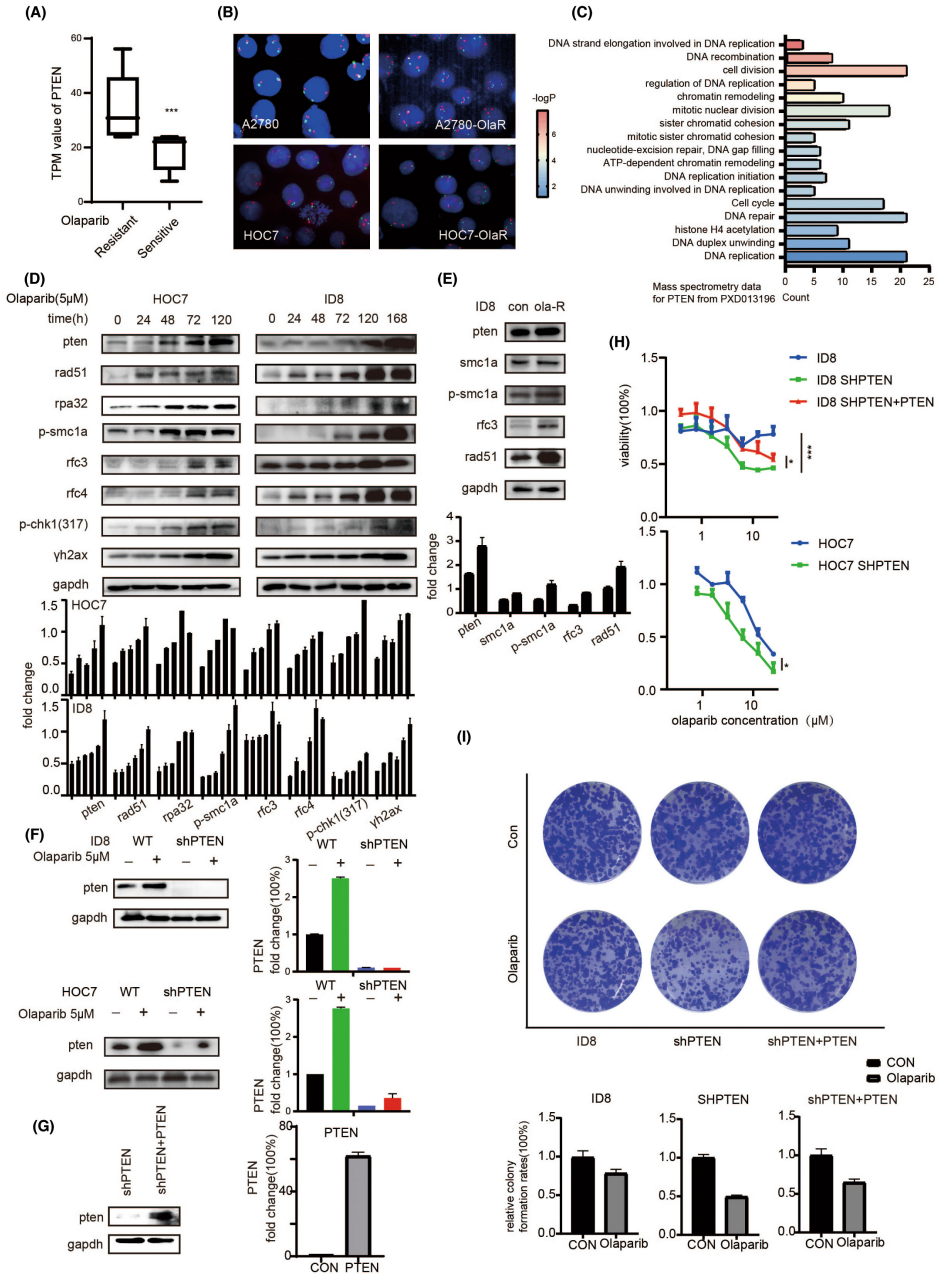
Expression of PTEN relates to olaparib resistance. (A) PTEN expression in RNA‐seq data of relative resistant and sensitive PDO models tested before. (B) FISH assay detecting the PTEN gene amplification of two olaparib‐resistant cell lines. Normal signal mode (two red and two green), PTEN gene amplification signal mode (mainly three red and three green). (C) The mass spectrum data of PTEN and proteins related to chromosome stability and DNA replication forks. (D) The change in protein expression showed by western blotting after olaparib treatment. (E) The change in protein expression showed by western blotting after acquired olaparib resistance. (F) The results of western blotting and Q‐RTPCR of PTEN after shRNA treatment. (G) The results of western blotting and Q‐RTPCR of PTEN after overexpression virus treatment.(H) The drug reactivity of olaparib was shown. *p*‐Value from Student *t*‐test: ns as nonsense, *p < 0.05, **p < 0.01, ***p < 0.001, ****p < 0.0001. (I) Representative images of the clonogenic assay in the presence of olaparib for 10 days.

Firstly, we analysed the difference between olaparib resistant and sensitive ovarian tumour tissues which were classified by PDO drug screening, we could see that PTEN changed significantly. The RNA‐SEQ data implied that the relative olaparib‐resistant group had a higher PTEN expression than the sensitive group (Figure 1A). At the genetic level, the signal mode detected by FISH assay also exhibited that PTEN had been amplificated after olaparib resistance occurred (Figure 1B). From the NCI‐60 tumour cell line data, which was analysed by the cell miner CDB website, it was found that the sensitivity of cells to olaparib was negatively correlated with PTEN expression (Figure S1A). The mass spectral data of PTEN also indicated that this protein was highly correlated with DNA replication and chromosomal stability (Figure 1C). The RFC and SMC protein families, which were overlapped between these two datasets, were related to olaparib resistance and affected by PTEN. Furthermore, pathway enrichment showed that pathways related to olaparib resistance were mainly correlated to DNA replication and chromosome stability. This result is consistent with the analysis of clustered regularly interspaced short palindromic repeats (CRISPR) screening data of acquired olaparib‐resistant cell lines in a public database (Figure S1B).

The results of the western‐blotting (Figure 1D) show that 5 μM olaparib treatment up‐regulated PTEN, together with phosphorylated checkpoint kinase 1 (p‐CHK1) and p‐replication protein A 32 kDa subunit (rpa32), indicating that the DNA replication function was activated by the stress induced by drugs. To maintain the structure and function of the heredity material, the corresponding proteins also increased. The increase in RFC and SMC protein family members could be regarded as a compensatory action for cells to cope with the actions of olaparib. We further explored this phenomenon and acquired olaparib‐resistant cell lines were successfully established (Figure S1C). The acquired olaparib resistant cell line also exhibited higher expression levels of these proteins (Figure 1E). Immediately afterwards, we used shRNA technology and overexpression viruses to up‐regulate or down‐regulate PTEN expression (shPTEN, shPTEN+PTEN) in ID8, and the expression levels of PTEN protein and mRNA were significantly changed (Figure 1F,G). Meanwhile, we found that the sensitivity to olaparib was enhanced after PTEN was downregulated, while after PTEN was restored in shPTEN cell lines, the sensitivity of olaparib decreased (Figure 1H,I). From the results above, we found that PTEN was essential in olaparib resistance.

### 3.2 AZD5153 reverse olaparib resistance by reducing PTEN expression

**FIGURE 2:**
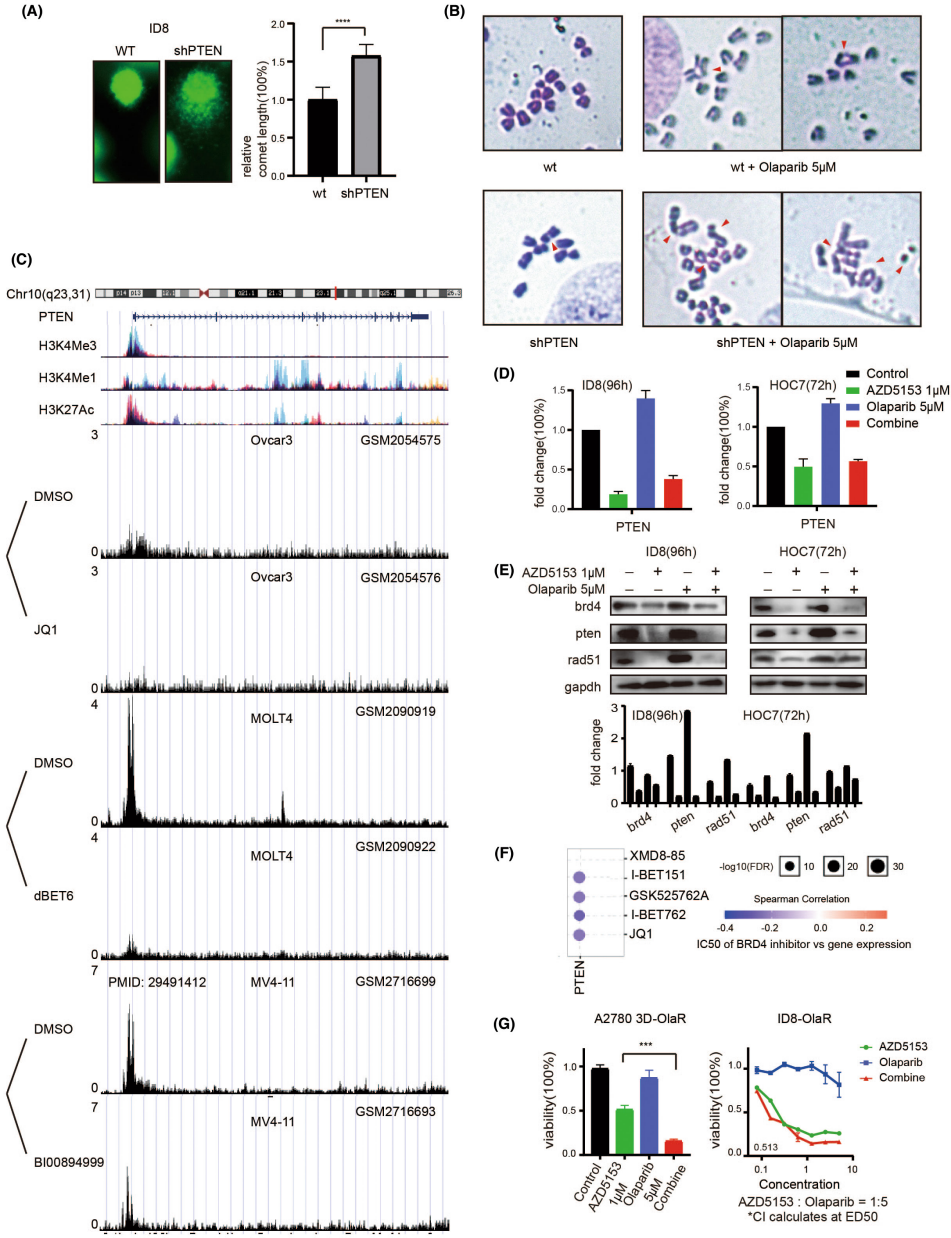
Reduced PTEN expression can reverse olaparib resistance. (A) Comet assay after shPTEN treatment. (B) The chromosome structural error after being treated by olaparib. (C) Chip‐seq data from the public database was shown in this figure. (D) The results of Q‐RTPCR of PTEN expression after olaparib and AZD5153 treatment. (E) The results of WB after olaparib and AZD5153 treatment. (F) According to CCLE, CTRP, and GDSC data, PTEN expression was negatively correlated with IC50. (G) The drug reactivity was calculated after acquired olaparib resistance.

We also compared the shPTEN and original parent cell lines, and the results showed that PTEN knockout triggered the endogenous DNA double strands break (Figure 2A). This phenomenon was observed using the comet assay and suggests that PTEN had a protective effect on the DNA. The examination of the chromosomes showed that olaparib treatment induced the repair of chromosomes in the original cell line, whereas those of the shPTEN cells exhibited complex aberrations at the same concentration of olaparib (Figure 2B and Figure S1F). This result indicated that PTEN knockout played an important role in the maintenance of DNA stability and chromosomal structure, which could sensitize ovarian cancer cells to olaparib. This is similar to the effect of AZD5153.^7^, ^25^ So, we assume that AZD5153 may affect the sensitivity of olaparib by reducing PTEN.

Subsequently, the chip‐seq data in the public database further verified the inhibitory effect of the BRD4 inhibitor on PTEN (Figure 2C). Tumour cells attempt to compensate for the stress caused by olaparib by increasing PTEN expression. AZD5153 specifically targets PTEN, causing dysfunction in DNA replication and chromosome stability, and thereby enhancing the sensitivity of cells to PARP inhibitors. Consistently, the effect of AZD5153 on PTEN was verified using quantitative reverse transcription‐polymerase chain reaction (qRT‐PCR), which showed the changes in PTEN expression levels in the cell line after drug treatment (Figure 2D). The western blotting results also showed that AZD5153 treatment inhibited PTEN expression in cells (Figure 2E).

The Gene Set Cancer Analysis (GSCA) database was used to analyse the protein expression and drug sensitivity data from Cancer Cell Line Encyclopedia (CCLE), Cancer Therapeutics Response Portal (CTRP), and Genomics of Drug Sensitivity in Cancer (GDSC) databases, and the results showed that the half‐maximal inhibitory concentration (IC_50_) of BRD4 inhibitors was negatively correlated with PTEN expression (Figure 2F). Therefore, it could be inferred that olaparib‐resistant cell lines with a higher PTEN expression would be more strongly affected by AZD5153 and be more sensitive to co‐treatment than those with lower PTEN levels. The reversal of olaparib resistance by AZD5153 could reflect the results under both 2D and 3D environments (Figure 2G).

### 3.3 AZD5153 and olaparib showed a widespread synergistic cytotoxicity in multiple ovarian cancer models

**FIGURE 3:**
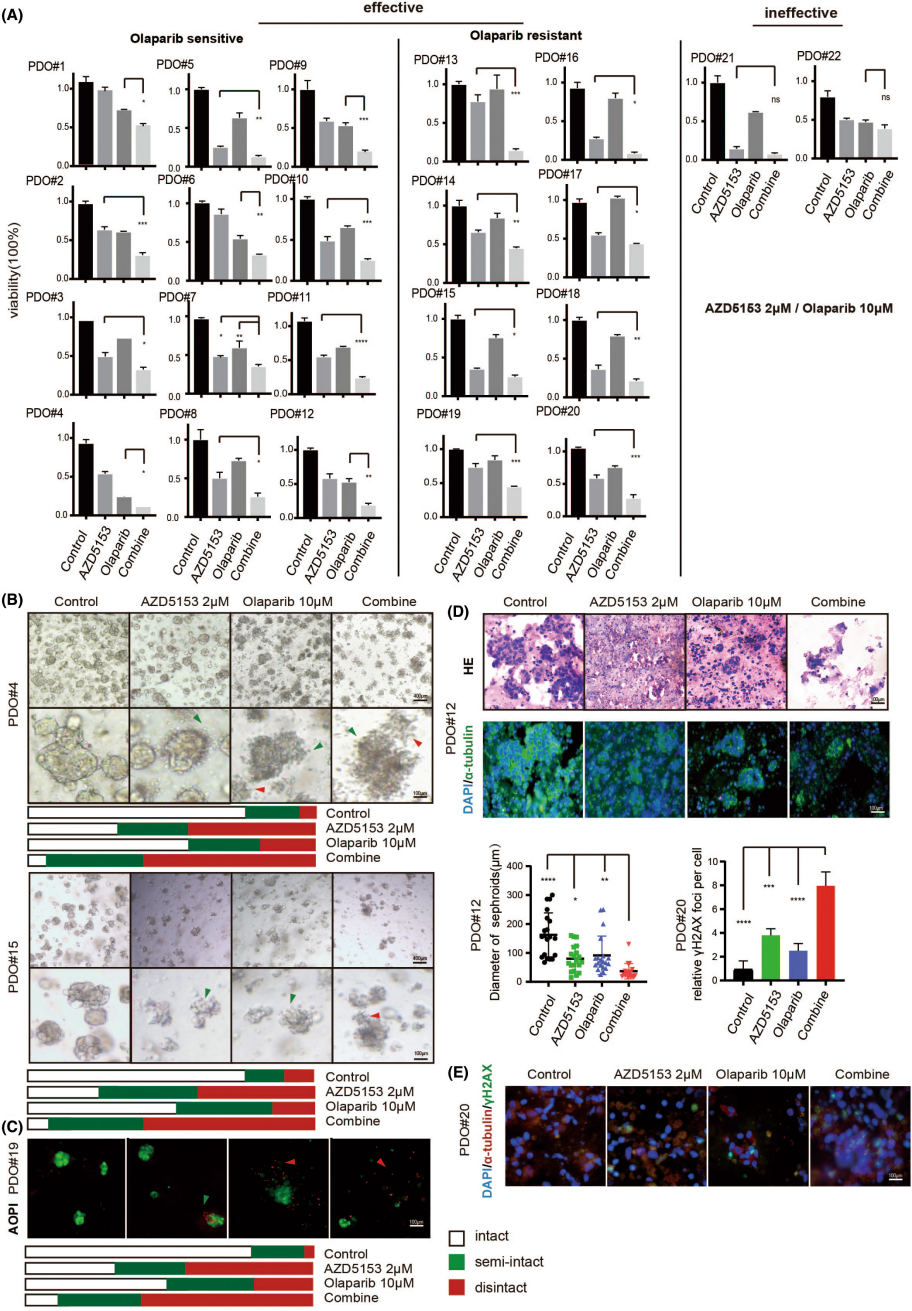
AZD5153 and olaparib have a synergistic cytotoxicity in multiple ovarian cancer models. (A) The figure showed the drug reaction of 22 PDO models. The results were divided into groups according to the relative sensitivity to olaparib. (B) The figure showed the spheroid made of cell line under the 3D culture condition by bright field of microscope. The picture below is an enlargement of the one above. The spheroids are classified into different status, which were signed by arrows in the enlargement picture. The percentage was shown below. (C) The figure showed the status of spheroids by AOPI staining, green light showed living cells, while the red light showed dead ones. The percentage of intact or semi‐intact spheroids are shown below. (D) The figure showed the size and density of PDO spheroids after slicing and staining. The diameter of spheroids was calculated. (E) The figure showed the change in γH2AX level after drug treatment.

In vitro ovarian cancer models, 15 cell lines and 22 PDO models (Table 1) were used to test the drug effects, and the result showed a marked synergistic anti‐tumour effect on both models (Figure 3A and Figure S2A). Among the experimental models with various genetic backgrounds, 86.7% (13/15) and 90.9% (20/22) of the cell lines and PDO models, respectively, were more sensitive to co‐treatment with olaparib and AZD5153 than either drug alone.

**TABLE 1:** “Clinical information of samples used in this study”.

PDO	Site	Age	Diagnosis	Response to olaparib
PDO#1	Ascite	53	High‐grade serous ovarian cancer	Sensitive
PDO#2	Ovary	57	High‐grade serous ovarian cancer	Sensitive
PDO#3	Ascite	57	High‐grade serous ovarian cancer	Sensitive
PDO#4	Ascite	63	High‐grade serous ovarian cancer	Sensitive
PDO#5	Ovary	58	High‐grade serous ovarian cancer	Sensitive
PDO#6	Ascite	56	High‐grade serous ovarian cancer	Sensitive
PDO#7	Ovary	47	High‐grade serous ovarian cancer	Sensitive
PDO#8	Metastasis	61	High‐grade serous ovarian cancer	Sensitive
PDO#9	Ascite	63	High‐grade serous ovarian cancer	Sensitive
PDO#10	Ovary	75	High‐grade serous ovarian cancer	Sensitive
PDO#11	Ascite	71	High‐grade serous ovarian cancer	Sensitive
PDO#12	Ovary	61	High‐grade serous ovarian cancer	Sensitive
PDO#13	Ovary	65	High‐grade serous ovarian cancer	Resistant
PDO#14	Ascite	47	High‐grade serous ovarian cancer	Resistant
PDO#15	Ascite	72	High‐grade serous ovarian cancer	Resistant
PDO#16	Ascite	66	High‐grade serous ovarian cancer	Resistant
PDO#17	Ascite	58	High‐grade serous ovarian cancer	Resistant
PDO#18	Malignant mesothelioma	61	Malignant mesothelioma	Resistant
PDO#19	Ovary	65	High‐grade serous ovarian cancer	Resistant
PDO#20	Ovary	49	High‐grade serous ovarian cancer	Resistant
PDO#21	Ovary	48	High‐grade serous ovarian cancer	Resistant
PDO#22	Ascite	54	High‐grade serous ovarian cancer	Resistant
PDX#1	Malignant mesothelioma	61	Malignant mesothelioma	
PDX#2	Ovary	62	High‐grade serous ovarian cancer	
PDX‐O#3	Ovary	46	Endometrioid ovarian cancer	

After calculating the cell viability, we used multiple methods to comprehensively examine the structure of the spheroid and observed that it was composed of obviously dead and depolymerized cells because of the cytotoxicity of the drugs (Figure 3B). Acridine orange and propidium iodide (AOPI) staining clearly showed that the red fluorescent‐labelled dead cells were gradually depolymerized from the PDO spheroid and scattered around the green fluorescent‐labelled live cells (Figure 3C). Haematoxylin and eosin (H&E) and fluorescence staining of the PDO model also showed a decrease in the spheroid diameter and increase in depolymerized cells (Figure 3D).

Furthermore, phosphorylated H2A.X variant histone (γH2AX) staining verified that cells in the PDO spheroid were killed by the drug co‐treatment (Figure 3E). In the co‐treated group, the dissociation of dead cells and decreased in diameter of the live PDO spheroids were obviously stronger than they were in the groups treated with either drug alone. Meanwhile, the combined therapeutic effect of AZD5153 and palbociclib in 2D cell lines were also synergistic lethality (Figure S2B,C). The synergistic effect was also examined in the A2780 cell line where we measured the response to AZD5153, olaparib, and their combination under 3D conditions (Figure S2D,E). While a published paper has confirmed that the repairing level was reduced,^26^ we find that the damage level increased in parallel. The results indicated that AZD5153 and olaparib showed a widespread synergistic cytotoxicity in multiple ovarian cancer models.

### 3.4 Co‐treatment with AZD5153 and olaparib damaged DNA by affecting its replication

**FIGURE 4:**
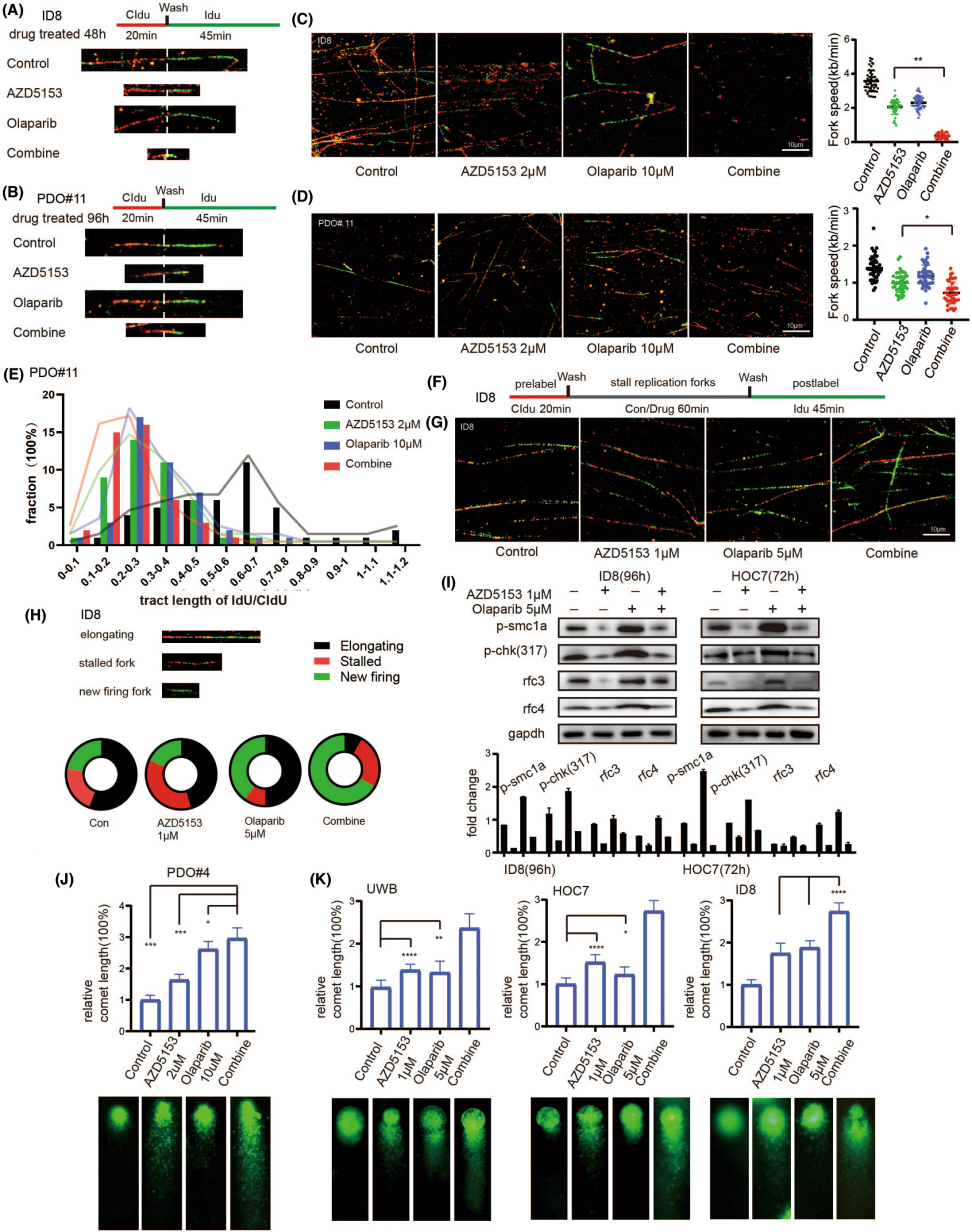
AZD5153 and olaparib can affect DNA replication in ovarian cancer. (A) The figure showed the sequence of drug treatment and fluorescence labelling of the ID8 cell line in DNA fibre assay. The drug was given before labelling. (B) The figure showed the sequence of drug treatment and fluorescence labelling of the PDO model. The typical status of DNA fibres in each group was also shown. (C) In the figure, we can see the typical DNA fibre status of the ID8 cell line in each group and the statistical results of replication speed under the influence of drugs. (D) In the figure, we can see the typical performance of PDO samples after drug treatment and the statistical results of replication speed in each group. (E) The statistical results of the replication fork stability represented by the ratio of fluorescence length of CIdU to IdU in the ID8 cell line. The distribution range of the IdU/CIdU ratio was obtained by counting more than 50 DNA fibres. (F) The sequence of drug treatment and fluorescence labelling of ID8 was shown. The drug was given between labelling. (G) The typical fluorescence type of each group in the DNA fibre test was shown in this figure. (H) The graph showed the typical status of DNA fibres. The proportion of different types of DNA in different drug treatment groups was also shown. (I) The picture showed the changes in proteins related to DNA replication fork stability and chromosome stability after the treatment of each group. (J) The figure showed the results of the comet assay after 96 h of drug treatment in the PDO model. (K) The result of comet assay demonstrated by three cell lines with different single drug sensitivity to olaparib or azd5153 was shown.

To further elucidate the mechanisms of combined lethal effect on AZD5153 and olaparib, we established two DNA fibre assays to examine the long‐ and short‐term drug effects on DNA replication. In the long‐term assay, we treated the ovarian cancer models for 2 (cell line) or 4 (PDO model) days before performing 5‐chloro‐2′‐deoxyuridine (CIdU) and 5‐Iodo‐2′‐deoxyuridine (IdU) labelling (Figure 4A,B). After drug treatment, measurement of labelled DNA fibres revealed a significant decrease in CIdU + IdU tract length in the ovarian cancer models (Figure 4C,D).

The tract length in the group co‐treated with both drugs was significantly shorter than it was in the groups treated with either drug alone. The calculation of replication rate^27^ also indicated that AZD5153 treatment slowed the fork progression rate of the DNA fibres, which was even slower in the co‐treated group than it was in the groups treated with either agent alone. The ratio of IdU to CIdU indicated a steady rate of DNA fork replication.^28^ The IdU/CIdU ratio decreased in the drug‐treated groups, especially in the co‐treated group, showing that the replication forks became unsteady and easier to degrade (Figure 4E) after treatment.

In the short‐time assay, the ovarian cancer cells were treated with drugs in between the CIdU and IdU labelling (Figure 4F). The condition of the IdU tracts indicated that the drugs immediately affected DNA replication. Furthermore, the results showed that the co‐treated group exhibited a typical signal pattern where the green fluorescence of the IdU tracts was much denser and shorter than those of the other groups (Figure 4G). The groups treated only with olaparib and only with AZD5153 exhibited a denser green signal and shorter IdU tracts, respectively, than those of the control group. We also identified the following three major patterns of fibre labelling, elongated, stalled and new firing (Figure 4H).^29^, ^30^

Olaparib treatment caused the development of more new firing DNA fibres,^31^ whereas AZD5153 produced more stalled fibres. Furthermore, in the replicating DNA fibres, AZD5153 treatment destabilized the forks and caused them to degrade in the early phase. The combined effects of both olaparib and AZD5153 caused more DNA fibres to break, and they were damaged further.

The change in the protein levels observed using western blotting (Figure 4I) verified the changes observed in the DNA fibre assay. Olaparib treatment up‐regulated proteins related to DNA replication stress and replication fork stability, whereas they were down‐regulated by AZD5153. The protein expression levels in the co‐treatment group suggested that the DNA fibres were in an unstable state. The disordered DNA replication resulted in strand breaks and the comet assay confirmed that the PDO model and cell lines showed more DNA double‐strand breaks after co‐treatment than they did following monotherapy with either drug (Figure 4J,K).

### 3.5 AZD5153 and olaparib can also cause greater damage in chromosome and lead to apoptosis

**FIGURE 5:**
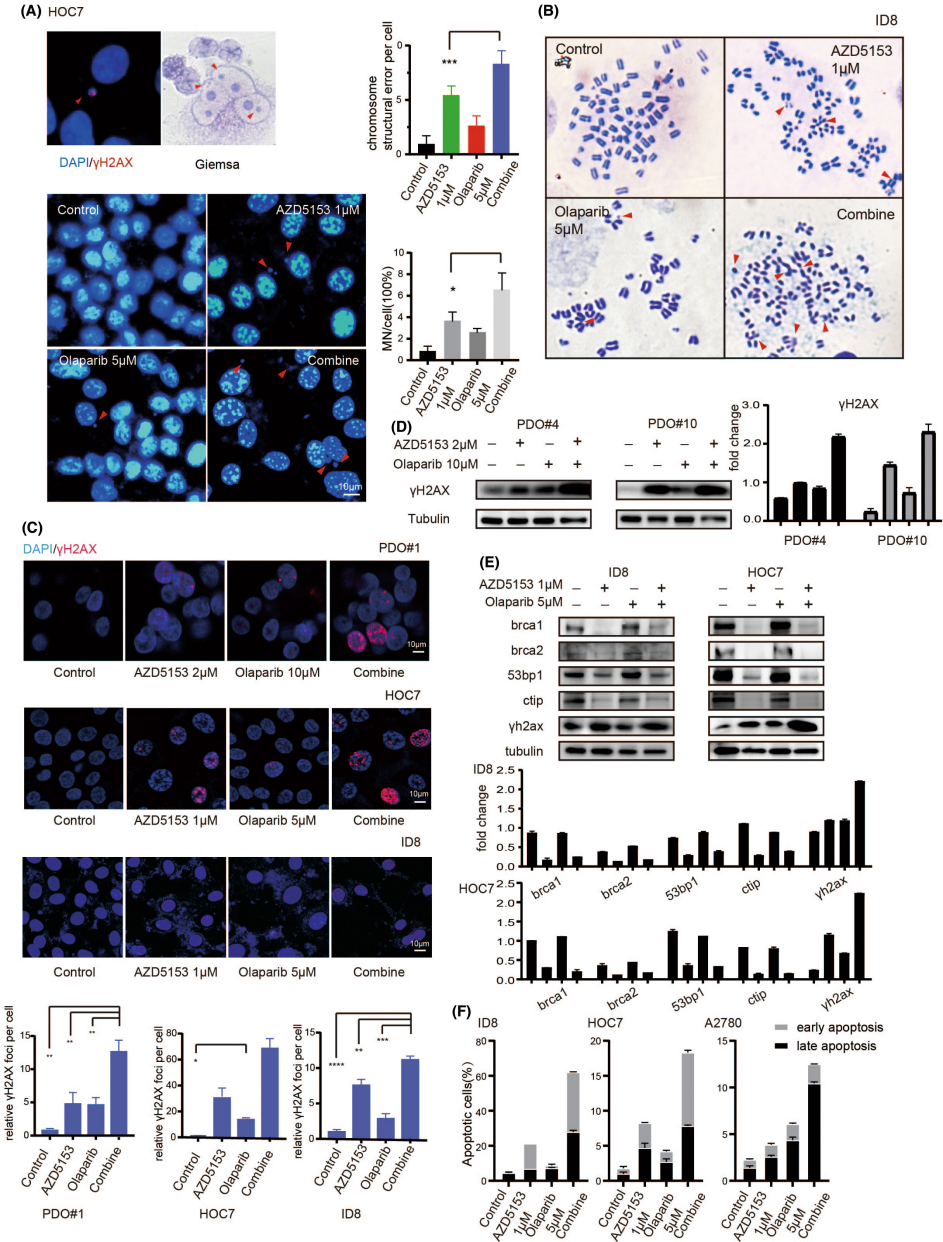
AZD5153 and olaparib cause chromosome damage and cell apoptosis. (A) The figure showed a typical micronucleus of the hoc7 cell line by DAPI/γH2AX staining and Giemsa staining. (B) The figure showed the results of the metaphase spread assay after the cell was treated with drugs. The arrow showed chromosome breakage and aberration. The percentage of chromosome structural error in each group after drug treatment was also shown. (C) The figure showed the change of γH2AX expression in cell lines and the PDO model by immunofluorescent. The relative γH2AZ foci per cell were also shown. (D) The figure showed the changes in protein expression of γH2AX. (E) The changes in protein expression which relates to DNA damage and repair in cell lines were shown. (F) The results of flow cytometry showed the percentage of apoptosis in cell lines after drug treatment.

We observed increased levels of micronuclei damage following co‐treatment with olaparib and AZD5153, which suggests a more severe chromosomal breakage (Figure 5A). We also investigated the chromosomal damage using a metaphase spread assay (Figure 5B) and the results showed that chromosomal fragments, breakage and aberration were higher in the co‐treated group than they were in the groups treated with either agent alone. This observation indicates that more damage occurred.

Cells with micronuclei and chromosomal breakage usually undergo apoptosis and we examined the cell status using immunofluorescence staining of the γH2AX foci (Figure 5C). The result suggested that more cells underwent apoptosis occurred after drug treatment, whereas the western blotting also showed similar changes in protein level (Figure 5D,E). Flow cytometry was also used to directly examine the apoptosis rate (Figure 5F). Collectively, these results suggest that co‐treatment induced greater cytotoxicity than treatment with either drug alone.

### 3.6 AZD5153 plus olaparib inhibit ovarian cancer growth in vivo

**FIGURE 6:**
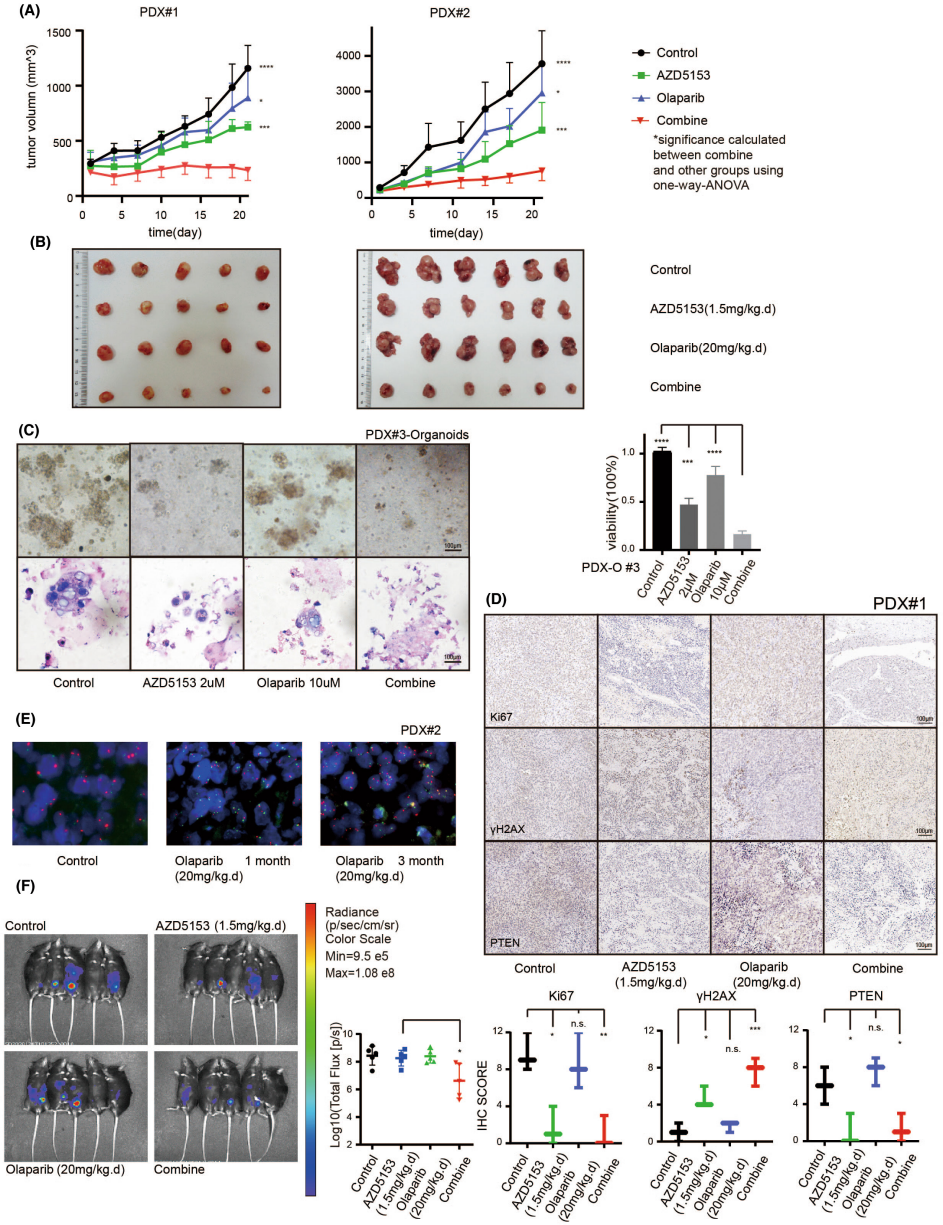
AZD5153 and olaparib inhibit ovarian cancer growth in vivo. (A) Two cases of PDX models which were divided into four groups were given vehicle, AZD5153, olaparib, and dual drugs, respectively. The dosage was shown in the figure. The curve of tumour volume in 21 days is also shown. (B) The figure showed the volume of tumour tissue which were taken after the mouse was sacrificed after drug treatment for 21 days. (C) After PDX‐O culturing and drug treatment, the size, and density of the spheroids in each group were shown in a bright field of microscope and by HE staining. The entire status of viability was calculated by CELLTiter GLO 3D, which was shown in the right. (D) Immunohistochemistry showed that the expression of Ki67, γH2AX, and PTEN in PDX#2 tumour tissue was changed after treatment. (E) The signal pattern of the FISH assay was performed before treatment, after 1 month, and after 3 months of olaparib treatment. (F) The figure showed the in vivo small animal imaging results of C57BL/6 mice planted by ID8 cells intraperitoneally and treated with drugs for 21 days.

Co‐treatment with AZD5153 and olaparib exhibited a similar synergistic effect in vivo. Both the patient and cell‐line‐derived xenografts showed a lower tumour burden after co‐treatment with both drugs. We used three different subcutaneous PDX BALB/C nu‐mouse models and one abdominal ID8‐derived xenograft C57BL/6 mouse model to analyse the combined drug effect. The tumour volumes of the subcutaneous PDX models were significantly lower after co‐treatment than after treatment with either drug alone (Figure 6A,B). The drug effect did not cause significant weight loss (Figure S3A).

After confirming the in vivo synergistic effect, we further tested whether the drug response was similar between the in vivo and in vitro models. First, a PDX‐Organoids model was used to examine the drug reactivity (Figure 6C) and the result showed that co‐treatment with AZD5153 and olaparib inhibited the spheroid growth, which was consistent with the findings in the PDX model. The immunohistochemistry results also showed changes in cell amplification and apoptosis that were similar to those observed in vitro (Figure 6D). Then, we treated one of the PDX models with olaparib for 3 months, during which we collected tumour samples for paraffin sectioning (Figure 6E). The FISH assay was used to amplify the *PTEN* gene over time and the results were consistent with those obtained in vitro.

C57bl/6 mice were intraperitoneally implanted with the ID8 ovarian cancer cell line and after 21 days of drug treatment, the tumour was measured using the in vivo small animal imaging technology (SIEMENS Inveon). The results showed a lower tumour burden in the co‐treated group (Figure 6F). This abdominal tumour model compensated for the deficiency in the nude mouse model, which was immune‐deficient. Both in vivo models produced the same result, which verified the synergistic effect of AZD5153 and olaparib in vitro.

## 4 DISCUSSION

In this study, we demonstrated the synergistic effect of AZD5153 and olaparib in multiple models and successfully used PDO models in experiments that are usually based on cell lines. The results indicated that the PDO models could be used to confirm the combined effect of AZD5153 and olaparib and further elucidate the mechanism of their synergistic action in ovarian cancer. The consistent results obtained in these models suggest the wide applicability of PDO and PDX model, which would allow exploration of the mechanisms of drug actions to be personalized. For patients with refractory ovarian cancer who do not benefit from most clinically used drugs, the discovery of personalized drug‐resistant mechanisms may facilitate the development of curative treatments.

The increase in DNA replication stress was confirmed to be affected by BRD4 and PARP inhibitors in a previous study.^11^ This difference in signalling pattern, where olaparib induces more new DNA fibre suggests that this agent triggered the increase of DNA replication stress,^32^ which stimulated the replication of more DNA fibres. These results confirm that the marked synergistic cytotoxicity was mediated by the instability induced in DNA fibres. The presence of micronuclei reflects damage to hereditary cellular material and the increase induced by co‐treatment with both agents was indicative widespread chromosomal breakage.

Our results indicated that phosphatase and tensin homologue (PTEN) was essential in the development of olaparib resistance. After olaparib treatment, ovarian cancer cells attempt to evade the lethal effect by enhancing the stability of genetic material including DNA and chromosomes by upregulating PTEN. This process can be prevented using AZD5153. Increased expression levels of PTEN were associated with a lower CI value, which reflects the strength of the effect of co‐treatment with both agents on PTEN expression. Consequently, these findings also suggested a stronger response to co‐treatment with AZD5153 and olaparib and that their synergistic effect was mediated by their combined effects on PTEN expression.

In addition, we encountered some unexplained challenges in the exploration of the mechanism underlying the synergistic effect of both agents. PTEN is well known to negatively regulate phosphoinositide 3‐kinase (PI3K) and the downstream AKT protein. However, as an inhibitor of BRD4, AZD5153 also strongly bind to enhancers^33^, ^34^ of various genes and decreases their expression, which could counter the antagonistic effect of PTEN and PI3K. A previous study suggested that co‐treatment with PARP and PI3K inhibitors exerts a stronger inhibitory effect on PTEN‐deficient cancer.^35^ In our study, AZD5153 reduced PTEN and PI3K simultaneously, which mimics the effect of PTEN deficiency and PI3K inhibition. We considered PTEN and not PI3K to be the key molecule because it is more strongly associated with the stability of hereditary material. Furthermore, the change in downstream RAD51 was consistent with that in PTEN.^36^ However, the downstream mechanisms mediating the role of PTEN still need further investigation.

This study had some limitations, which are worth mentioning. Organoid cultures are easier to establish using more malignant tumours,^37^ mainly because they have a lower level of differentiation and higher stemness than less malignant tumours. During model establishment, we obtained a higher success rate with high‐grade serous ovarian cancer than we did with other less virulent samples, which may have caused selection bias. To avoid the influence of this selection bias, we used multiple models to confirm our conclusions. During the experiment, we also found that even with the same patient, differences occurred in drug responses. The drug responsiveness led us to infer that tumour cells isolated from ascites had the highest activity and drug resistance, and this was likely because they had an innate ability to survive as spheroids.^38^ However, the reason for the higher drug resistance of tumour cells in ascites has not been fully elucidated yet.

In conclusion, we present strong evidence supporting the notion that AZD5153 and olaparib have a widespread synergistic effect. Olaparib treatment upregulates PTEN, and then DNA and chromosome stability will rise, which made ovarian cancer acquire drug resistance. AZD5153 sensitizes cells to olaparib and reverses the acquired resistance by down‐regulating PTEN expression levels to destabilize hereditary materials. In this study, we used the following multiple ovarian cancer models PDX, PDO and 3D/2D cell lines to elucidate the co‐effect of AZD5153 and olaparib in vivo and in vitro. The similar results of these models further proved that the mechanism identified was consistent with the biological process occurring in ovarian cancer patients after drug treatment. This, consistency between the results of different models suggests the possibility of translating these laboratory research findings into clinical studies towards developing treatments.

## AUTHOR CONTRIBUTIONS

**Yuhan Huang:** Investigation (equal); methodology (equal); project administration (equal); writing – original draft (equal). **Chen Liu:** Data curation (equal); formal analysis (equal); software (equal). **Lixin You:** Supervision (equal); validation (equal). **Xi Li:** Supervision (equal); validation (equal). **Gang Chen:** Validation (equal); visualization (equal). **Junpeng Fan:** Conceptualization (lead).

## CONFLICT OF INTEREST STATEMENT

We confirm that this work is original and has not been published elsewhere, nor is it currently under consideration for publication elsewhere. We declare that there are no competing interests.

## Supporting information


Figure S1.

Figure S2.

Figure S3.

## Data Availability

All data generated or analyzed during this study have been included in the article. Additionally, the raw data that support the findings of this study are available from the corresponding author upon reasonable request.
