# Supplementary figures and images for: Synergistic effect of PARP inhibitor and BRD4 inhibitor in multiple models of ovarian cancer
Source: J Cell Mol Med. 2023 Feb 8;27(5):634–49. doi: 10.1111/jcmm.17683 (PMC9983312; doi:10.1111/jcmm.17683)

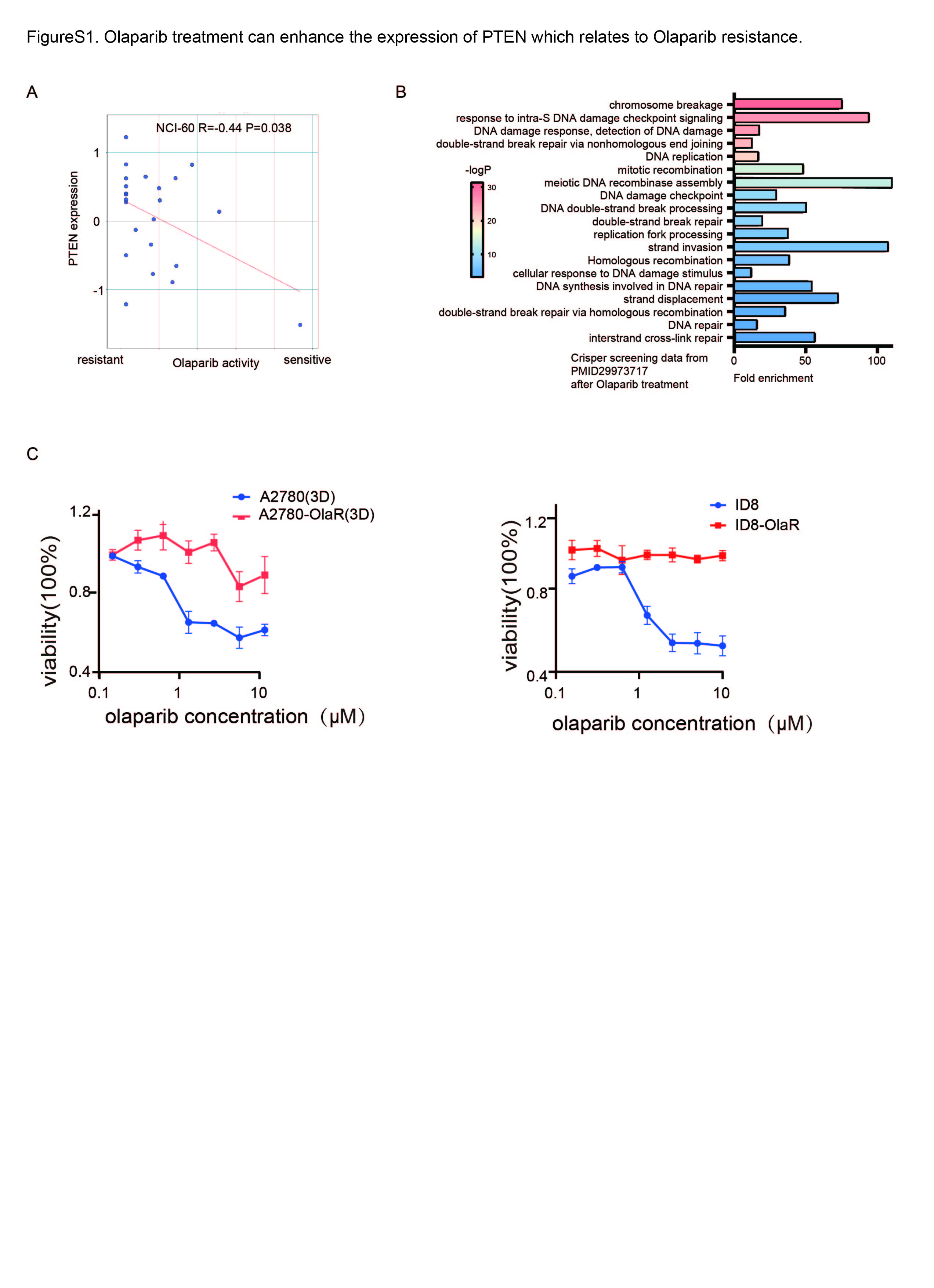

Supplement: Supplementary file 1 — Figure S1. [file JCMM-27-634-s002.jpg]

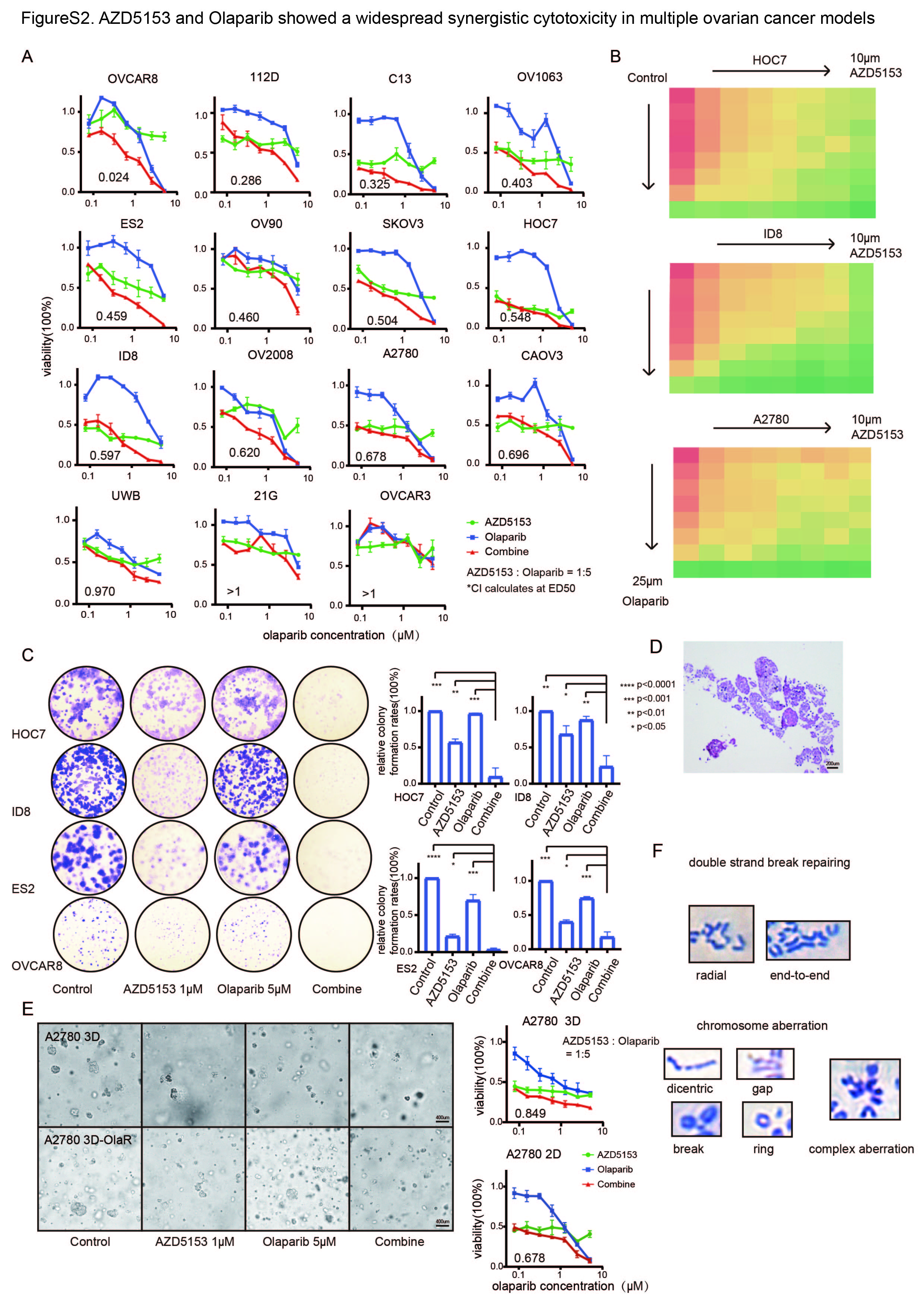

Supplement: Supplementary file 2 — Figure S2. [file JCMM-27-634-s001.jpg]

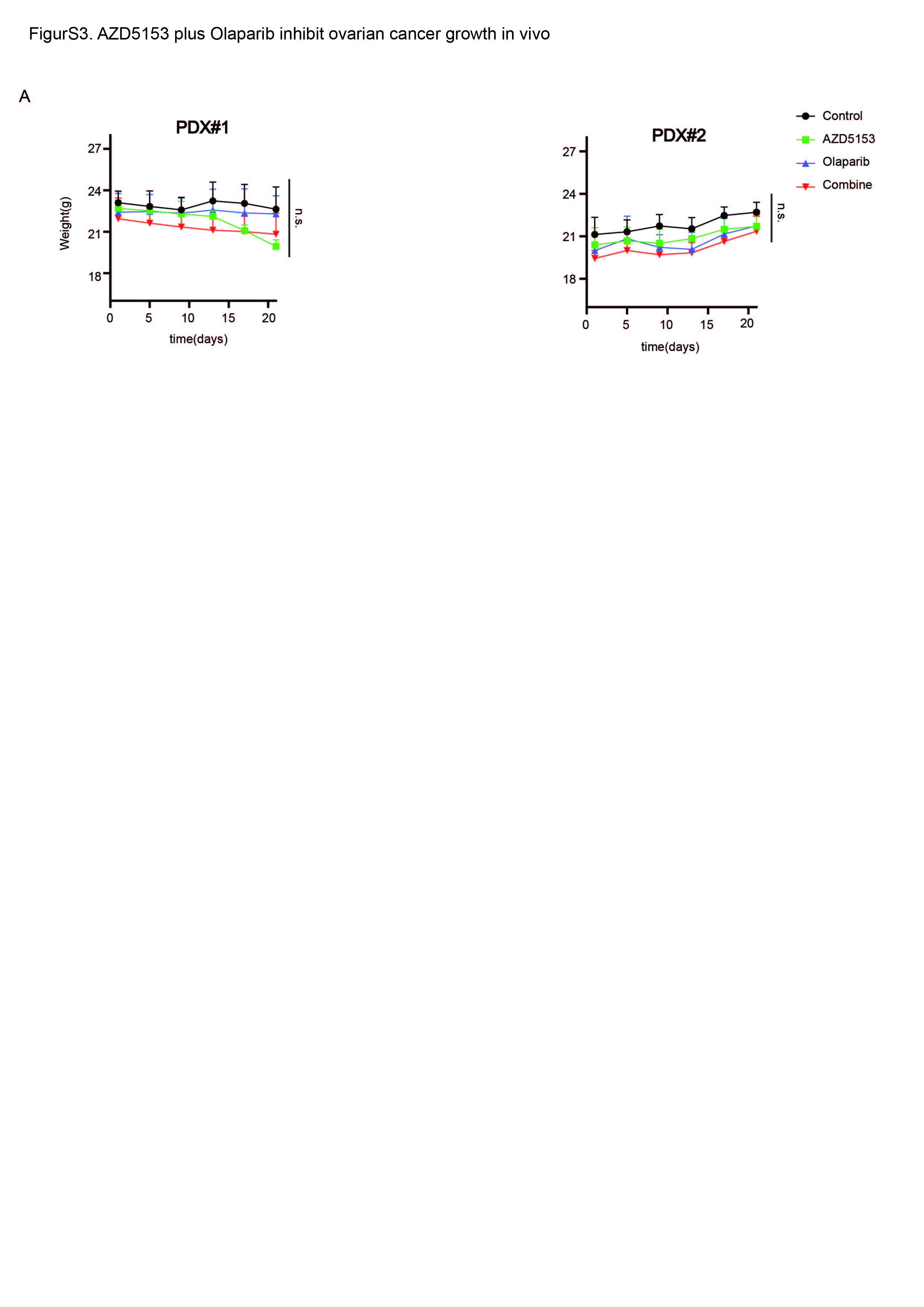

Supplement: Supplementary file 3 — Figure S3. [file JCMM-27-634-s003.jpg]
